# Two-stage pancreatic head resection after previous damage control surgery in trauma: two rare case reports

**DOI:** 10.1186/s12893-020-00763-2

**Published:** 2020-05-12

**Authors:** Jorge Paulino, Emanuel Vigia, Miguel Cunha, Edgar Amorim

**Affiliations:** 1 Centro Hepatobiliopancreático e de Transplantação, Centro Hospitalar Universitário de Lisboa Central, Hospital Curry Cabral, Universidade Nova de Lisboa, Lisboa, Portugal; 2 Department of Surgery, Centro Hospitalar Universitário do Algarve – Unidade de Portimão, Portimão, Portugal

**Keywords:** Pancreatic trauma, Pancreaticoduodenectomy, Pancreatic injury, Duodenal trauma, Case reports

## Abstract

**Background:**

This study describes the successful treatment of two clinical settings of grade V pancreaticoduodenal blunt trauma only possible due to the prompt collaboration of a peripheral trauma hospital and a central hepatobiliary and pancreatic unit.

**Case presentation:**

We reviewed the clinical records of two male patients aged 17 and 47 years old who underwent a two-stage pancreaticoduodenectomy after a previous Damage-Control Surgery (DCS). Both patients were transferred to our Hepatobiliopancreatic Unit 2 days after immediate DCS with haemostasis, debridement, duodenostomy, gastroenterostomy, external drainage and laparostomy. One day after, they both underwent a two-stage Whipple’s procedure with external cannulation of the main bile duct and the main pancreatic duct with seized calibre silicone drains through the skin. The reconstructive phase was performed two weeks later. The first patient had an uneventful post-operative course and was discharged on post-operative day 8. The second patient developed a high debt biliary fistula on post-operative day 5 being submitted to a relaparotomy with extensive peritoneal lavage. After conservative measures the fistula underwent a progressive closure in 15 days, and the patient was discharged at post-operative day 50 without any limitations.

**Conclusions:**

Pancreaticoduodenectomy is a life-saving operation in selected grade V pancreaticoduodenal trauma lesions. DCS is a salvage approach, often performed in peripheral hospitals, making an early referral to an hepatobiliopancreatic centre mandatory to achieve survival in these severely injured patients. A two-staged Whipple’s operation for severe duodenal / pancreatic trauma can be performed safely and may represent a life-saving option under these very unusual circumstances.

## Background

Duodenal and pancreatic injuries are very rare compared with those of other abdominal organs. Isolated duodenal injuries have an estimated 4.3% incidence in all abdominal injuries, and pancreatic trauma occurs in only 3% [1, 2]. By its protected retroperitoneal location, an excessive blunt or penetrating trauma is required to be able to injury both the pancreas and the duodenum. This is the main cause to the high rates of associated abdominal injuries under those circumstances [3].

The most common injury is at the neck of the pancreas, by direct compression against the vertebral column, while less commonly, pancreas head or tail injuries may develop due to blows to the flanks [4].

Hemodynamically unstable patients need to have an immediate surgical exploration, often with a Damage-Control Surgery (DCS), and the reconstructive interventions should be planned at a later stage, when the potentially lethal factors (haemorrhage, shock, peritonitis, acidosis, hypothermia or coagulopathy) are treated efficaciously and the patient is stabilized [5].

However, many complex injuries occur far from differentiated medical centres, and the first approach to these complex patients is made in peripheral hospitals, lacking surgeon and institutional experience, making crucial an early referral collaboration [6]. Under these circumstances pancreaticoduodenectomy is a very uncommon procedure, and a two-stage operation may be considered if the surgeon is dealing with an unstable patient [7, 8]. Moreover, this approach is usually reserved for the most severe injuries of both the head of the pancreas and the duodenal arch, grade 4 or 5 according with the organ injury scale and the American Association for the Surgery of Trauma [9, 10] (Table 1).

**Table 1:** Organ injury scale of the American Association for the Surgery of Trauma

Injured structure and AAST grade	Characteristics of injury	AIS − 2005 score
**Pancreas**		
I	Small hematoma without duct injury; superficial laceration without duct injury	2
II	Large hematoma without duct injury or tissue loss; major laceration without duct injury or tissue loss	2
III	Distal transection or parenchymal laceration with duct injury	3
IV	Proximal transection or parenchymal laceration involving ampulla	4
V	Massive disruption of pancreatic head	5
**Duodenum**		
I	Single-segment hematoma; partial-thickness laceration without perforation	2
II	Multiple-segment hematoma; small (\50% of circumference) laceration	2
III	50–75% Disruption (laceration) of segment D2 or 50–100% disruption of segment D1, D3, or D4	3
IV	Large (75–100%) laceration of segment D2; rupture of ampulla or distal CBD	4
V	Massive duodenopancreatic injury; devascularization of duodenum	5

Scores were collected from the organ injury scale of the American Association for the Surgery of Trauma [9] and the Abbreviated Injury Scale (AIS 2005) [10]

Here we present two cases of severe blunt duodenal / pancreatic trauma, using this two-stage approach after a previous DCS. Both patients had been managed previously with haemostatic procedures followed by gastroenterostomy before being referred to our institution for definitive surgery.

## Case presentation

### First clinical setting

A 17-year-old male suffered a severe motorcycle accident with a deep upper abdominal blunt trauma. Soon after the accident he was resuscitated and taken for emergency laparotomy at the nearest Trauma Centre. A massive right-sided retroperitoneal hematoma was detected, with active haemorrhage from a severely disrupted pancreatic head, duodenum and lower biliary tract. An extended Kocher manoeuvre was performed while compressing the hepatoduodenal ligament for haemostasis. At that time a main pancreatic duct disruption was demonstrated, without any evidence of the portal or the superior mesenteric vein involvement. The patient was hypothermic and moderately acidotic, fostering a DCS. After multiple sutures around the avulsion areas, the active bleeding was stopped and a cholecystectomy, complemented with a silicone T-tube drainage of the main bile duct and a gastroenterostomy were performed, as well as a duodenostomy with a Foley catheter. Two large silicone drains were placed and the patient was transferred to Lisbon by plane, with a laparostomy.

The patient was admitted in the Intensive Care Unit (ICU) of Curry Cabral Hospital where supportive care was initiated. Improvement of physiological parameters and hemodynamic stability was achieved in 48 h, despite a progressive biliary drainage from the silicone rubber drains.

**Fig. 1:**
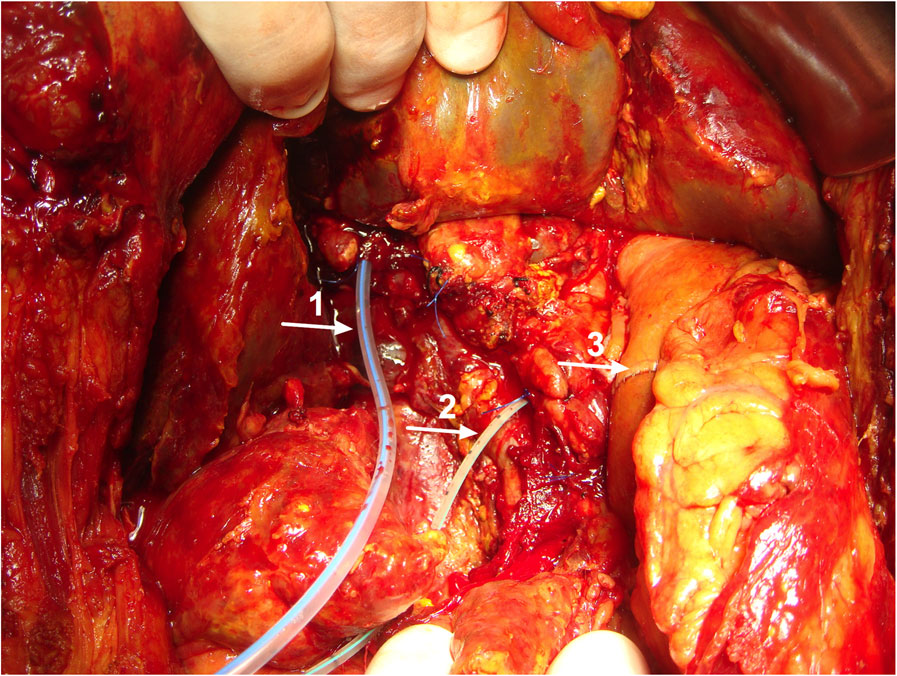
Resection of the pancreatic head and duodenum. Resection was performed and the silicon endoluminal catheters have been secured in place with nylon sutures and are indicated by arrows: 1 - endoluminal catheter in main bile duct; 2 - endoluminal catheter in main pancreatic duct; 3 – gastrojejunostomy

A second surgery was then decided, revealing bile staining throughout the entire peritoneal cavity, revealing slight oedema of the bowel loops without contamination. A Whipple’s resection was performed, exposing a main bile duct of around 5 mm diameter and a centred and easily located 3 mm main pancreatic duct: they were both cannulated with seized calibre silicone drains exiting the abdomen through the skin. Before closing the abdomen both catheters were secured to the surrounded parenchyma with a nylon transfixed suture, and the right upper quadrant was drained with two closed suction systems (Fig. 1).

The post-operative course was uneventful. Fourteen days later the reconstructive operation was performed without any unusual factors, and a classical Whipple operation was completed after removal of the well-placed silicone endoluminal catheters. Again, the post-operative course was uneventful and the patient was discharged from the hospital at post-operative day 8.

### Second clinical setting

**Fig. 2:**
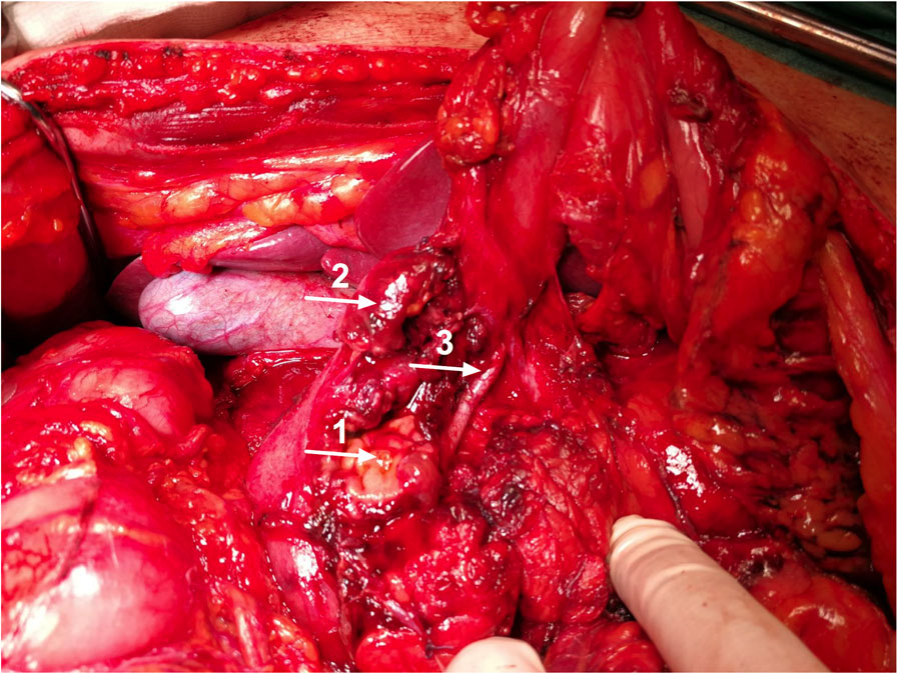
View after haemostasis, showing a more than 75% rupture of the second duodenum (arrow 1), massive disruption of pancreatic head (arrow 2) and denudation of the main bile duct (arrow 3)

**Fig. 3:**
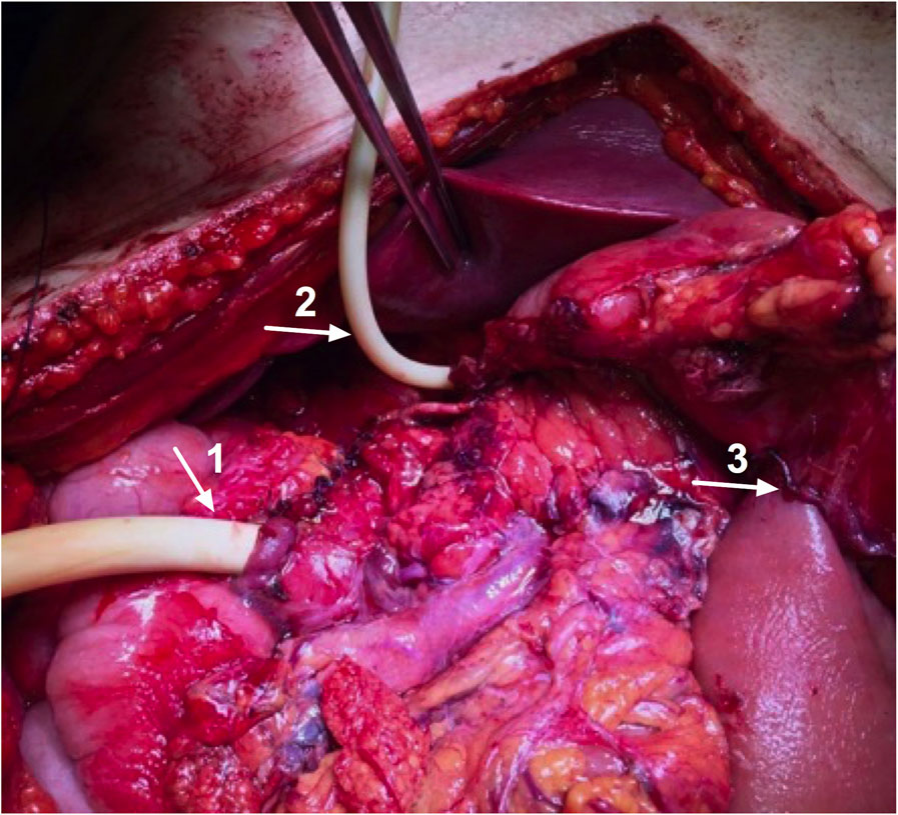
View after resection of the D1 and part of D2, tube duodenostomy (arrow 1), showing the T tube in the common bile duct (arrow 2) and gastroenterostomy (arrow 3)

This patient was a 47-year-old male paddle-surfer who suffered a severe deep and blunt trauma in the upper abdomen with his paddle-surf board. He was immediately transported to the nearest emergency room (Trauma Centre of Portimão Hospital), where he was submitted to a first medical evaluation. At that time, he was hemodynamically stable but with severe abdominal tenderness and acute abdomen signs. An abdominal CT scan revealed a haemopneumoperitoneum with distinctive evidence of pancreatic contusion in the uncinated process and duodenal rupture. No main pancreatic duct disruption was shown. He was then sent to emergency surgery and a median laparotomy was performed. There was a 300 cc haemoperitoneum with its origin in the duodenum and the head of the pancreas, but as in the first clinical setting, there was no lesion detected in the portal or the superior mesenteric vein. A more than 75% rupture of the second duodenum was confirmed, with alleged bile duct integrity and a complete main pancreatic duct disruption in the neck (Fig. 2). A DCS was performed, with haemostasis, a duodenostomy with a Foley catheter, a silicone T-tube drainage of the main bile duct (after a normal cholangiography), cholecystectomy and a gastroenterostomy (Fig. 3). Again, two large silicone rubber drains were placed and the patient was transferred by plane to the HPB Unit of Curry Cabral Hospital, with a laparostomy.

The patient was admitted in the ICU of Curry Cabral Hospital, obtaining an improvement of physiological parameters and achieving hemodynamic stability in 48 h, once again with a progressive drainage rich in amylase from the silicone rubber drains.

**Fig. 4:**
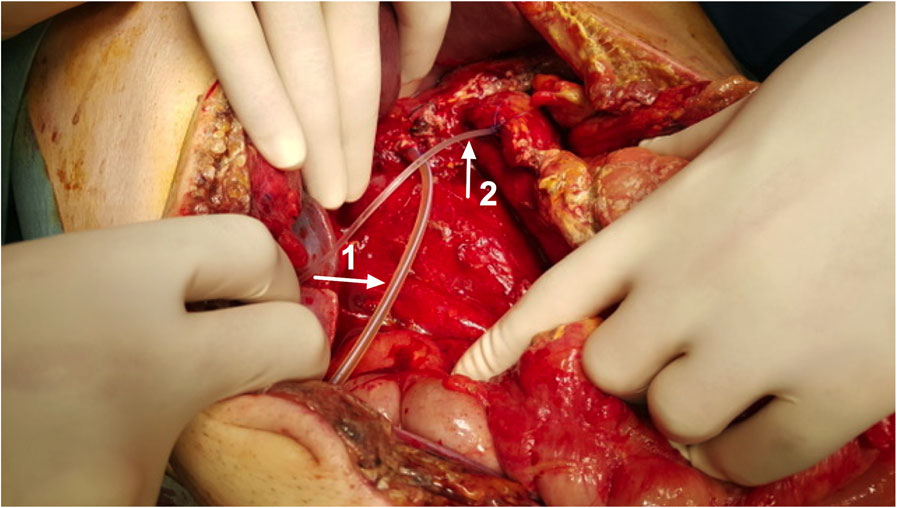
View after the resection, showing both catheters secured to the surrounded parenchyma with a nylon transfixed suture, indicated by arrows: 1 - endoluminal catheter in main bile duct; 2 - endoluminal catheter in main pancreatic duct

As the drainage turned biliary, a second surgery was then decided, confirming bile content throughout all the peritoneal cavity, with considerable oedema of the bowel loops. A Whipple’s resection was performed, exposing a main bile duct of around 4 mm diameter and a centred and easily located 3 mm main pancreatic duct with a normal consistency parenchyma. The same surgical protocol was used, cannulating both ducts with seized calibre silicone drains exiting the abdomen through the skin (Fig. 4).

Again, both catheters were secured to the surrounded parenchyma with a nylon transfixed suture, and the right upper quadrant was drained with two closed suction drains. The abdomen was then closed.

The post-operative course was uneventful. Sixteen days later, the reconstructive operation was performed just as in the previous case, and a classical Whipple was completed after removal of the well-placed silicone endoluminal catheters.

The post-operative course was uneventful until the 5th day. At this point, there was progressive abdominal pain and a high debt biliary fistula, both from the drain and the abdominal wall. A relaparotomy was then performed, with extensive peritoneal lavage, confirming an impenetrable and sealed upper abdominal compartment: the suction drains were changed and the laparotomy was closed with separated full-thickness stiches.

After conservative measures and serial image control, the fistula underwent a progressive closure in 15 days, and the patient was discharged from the hospital at post-operative day 50. He reported returning to his normal lifestyle without any limitations.

## Discussion and conclusions

Whipple’s procedure in trauma-related circumstances is a very unusual procedure. This kind of pancreatic resection was reported for the first time in literature for trauma patients in the 60s [11, 12]. This is due to the very low incidence of duodenal and pancreatic injuries compared to other abdominal organs (respectively 4.3 and 2.0% of all abdominal injuries) [13–15], and to the very high mortality rate ranging from 20 to 100% [16, 17].

The two cases described were both grade V, making a surgical approach inevitable. Nonoperative management would be inappropriate in these high-grade injuries, as it has been demonstrated in retrospective studies [18, 19]. This is not an absolute truth since non-operative treatment for severe lesions is possible as demonstrated by Wilden in one of the largest retrospective studies [20]. However, in these two cases the presence of both extensive trauma to the head of the pancreas and severe combined pancreaticoduodenal injury justified the operative decision [21]. The clinical and geographic circumstances determined the option of performing DCS as the first approach [22, 23]. This strategy is indicated with acidosis (pH < 7.3), hypothermia (temperature < 35 °C) and coagulopathy (non-mechanical bleeding) [24].

Due to its rarity, the application of DCS techniques in relation to pancreatic and duodenal injuries is hardly ever described in literature, aiming at preventing further haemorrhage or abdominal contamination with bile and pancreatic juices [25]. Packing combined with adequate pancreatic and duodenal drainage, as in the primary approach of these two cases, was the appropriate DCS technique under those circumstances [26]. The need for total pancreatectomy is exceptional during DCS, under these circumstances. However, it might be considered only as a definitive procedure, if the extent of debridment required left no other choice. This difficult decision might be taken only by a center specialized in pancreatic surgery.

Also, attempting a Whipple’s resection on a coagulopathic, hypothermic and acidotic patient, is considered very risky, assuming that a higher experience / better outcomes relation is also true in unstable trauma patients [20].

Additionally, the status of the main pancreatic duct as well as the global status of the patient determinate the management of pancreatic injuries. Screening trauma patients for pancreatic injury using CT scan must be cautious since the sensitivity is reported as poor, as in the second clinical case [27]. A careful inspection and a bimanual palpation of the pancreas should be enough to demonstrate a main duct injury, avoiding added risk by other manoeuvres such as intraoperative pancreatography [28].

In most cases described in literature, the second and definite surgery is performed in approximately 48 h, allowing appropriate staff and patient conditions. In the two cases described, however, the second surgery has been precipitated by the aggravation of the abdominal conditions, namely the bile content throughout the whole peritoneal cavity. The high amylase content of the drainage activated the bile leaking from the injured duodenum, and led to a pancreatitis-like reaction and to a considerable oedema of the bowel loops, which, altogether, precluded the immediate reconstruction after pancreaticoduodenectomy. One might argue why deciding to postpone the definitive reconstruction, as it was taking place at an experienced hospital, with an experienced pancreatic team. There is evidence that, performing anastomosis despite the unfavourable presented circumstances is as safe as delaying it into a two-stage procedure (38.7% vs 34.2% mortality) [29]. Nevertheless, assuming such a high late mortality rate, mostly due to multiple organ failure and sepsis [30] discouraged us to do so, particularly after an open abdomen scenario.

The appropriate conditions of both the common hepatic duct and the main pancreatic duct made safe and possible to cannulate both with separate silicon catheters, allowing a total and separated external drainage of bile and pancreatic content. Both drainages were left in place in a good position, allowing local conditions for the final surgical step.

The length of time until the anastomotic surgery (15 days) was decided based on the stabilization of the clinical situation of the patient. Although this could be considered a late timing since it might correspond to a high peritoneal inflammatory reaction after a biliary peritonitis, it has been reported that in abdominal sepsis caused by secondary peritonitis, the acute phase proteins synthesis stimulated by cytokines recover to normal values only after day 12 of the post-operative period [31]. The fact that, in both cases, there were no inaccessible exsanguinant lesions involving both the retropancreatic portal nor the superior mesenteric vein, allowed this 15-day delay between the two stages of pancreatoduodenectomy; under those circumstances, that period would have been considered excessive [32]. Moreover, there is no recommendation in literature concerning the timing of the definitive procedure. On the second patient, we repeated the surgical protocol previously used and based on the excellent outcome of the first clinical setting. The significant morbidity (biliary fistula) reported in the second clinical setting may reflect the inappropriate judgement of the optimal local conditions after a pancreatitis-like reaction. There are no recommendations found whatsoever, concerning the optimal approach of pancreatic and duodenal grade V injuries due to its rarity. Most publications are based on case reports, like ours, making it difficult to interpret the scarce literature available [33].

Pancreatic and duodenal trauma remains a clinical challenge, with a high mortality rate. Grade V injuries are difficult to deal with, as they occur very rarely, lacking high level evidence based recommendations in the literature. DCS definitely changed the references for these severe cases [34], but the mortality remains high, with most deaths occurring before a definitive management [33].

Early diagnosis remains problematic, especially following blunt abdominal trauma. The presence of main pancreatic duct injury is one of the major determinants of both morbidity and treatment decisions.

Trauma Whipple procedure may be inevitable in the sequence of previous DCS, and it should always be decided after early patient referral to an experienced hepatobiliary and pancreatic centre, avoiding inappropriate aggressive or conservative indications.

In conclusion, reconstruction after a pancreaticoduodenectomy for duodenal / pancreatic trauma can be performed safely as a two-staged procedure and may represent a life-saving option under these very unusual circumstances.

## Data Availability

The datasets generated and/or analysed during the current study are not publicly available due to protecting individual patient privacy but are available from the corresponding author on reasonable request.

## Abbreviations

CT: Computed tomography
DCS: Damage-Control Surgery
HPB: Hepatopancreatobiliary
ICU: Intensive Care Unit
